# Tumor-Intrinsic Nuclear β-Catenin Associates with an Immune Ignorance Phenotype and a Poorer Prognosis in Head and Neck Squamous Cell Carcinomas

**DOI:** 10.3390/ijms231911559

**Published:** 2022-09-30

**Authors:** Mario Sánchez-Canteli, Luis Juesas, Irati Garmendia, María Otero-Rosales, Alfonso Calvo, Monica Alvarez-Fernández, Aurora Astudillo, Luis M. Montuenga, Juana M. García-Pedrero, Juan P. Rodrigo

**Affiliations:** 1 Department of Otolaryngology, Hospital Universitario Central de Asturias, Instituto de Investigación Sanitaria del Principado de Asturias (ISPA), Instituto Universitario de Oncología del Principado de Asturias, University of Oviedo, 33011 Oviedo, Spain; 2 Centro de Investigación Biomedica en Red de Cáncer (CIBERONC), 28029 Madrid, Spain; 3 Center for Applied Medical Research (CIMA), Department of Pathology, Anatomy and Physiology, University of Navarra and Navarra’s Health Research Institute (IDISNA), 31008 Pamplona, Spain; 4 Tumor Biobank, Instituto de Investigación Sanitaria del Principado de Asturias (ISPA), Instituto Universitario de Oncología del Principado de Asturias, University of Oviedo, 33011 Oviedo, Spain

**Keywords:** head and neck, squamous cell carcinoma, β-catenin, tumor infiltrating lymphocytes, PD-L1

## Abstract

Activation of WNT/β-catenin signaling has been associated with a non-T-cell-inflamed tumor microenvironment (TME) in several cancers. The aim of this work was to investigate the relationship between β-catenin signaling and TME inflammation in head and neck squamous cell carcinomas (HNSCCs). Membrane and nuclear β-catenin expression, PD-L1 expression, and CD8+ tumor-infiltrating lymphocyte (TIL) density were jointly evaluated by immunohistochemistry in a series of 372 HPV-negative HNSCCs. Membrane β-catenin levels decreased in carcinomas compared to the normal epithelium. Positive nuclear β-catenin was detected in 50 tumors (14.3%) and was significantly associated with a low CD8+ TIL density (168 cells/mm^2^ versus 293 cells/mm^2^ in nuclear-β-catenin-negative cases; *p* = 0.01) and a tendency for a lower expression of PD-L1, resulting in association with a noninflamed TME (i.e., type II, immunological ignorance). Multivariate Cox analysis further demonstrated that low infiltration by CD8+ TILs (HR = 1.6, 95% CI = 1.19–2.14, *p* = 0.002) and nuclear β-catenin expression (HR = 1.47, 95% CI = 1.01–2.16, *p* = 0.04) were both independently associated with a poorer disease-specific survival. In conclusion, tumor-intrinsic nuclear β-catenin activation is associated with a non-inflamed TME phenotype and a poorer prognosis, thereby suggesting a possible implication as an immune exclusion mechanism for a subset of HNSCC patients.

## 1. Introduction

Head and neck squamous cell carcinoma (HNSCC) is the sixth most commonly diagnosed cancer and the most frequent malignancy in the head and neck region [1]. HNSCC is a group of heterogeneous diseases that includes tumors arising at various anatomical locations (oral cavity, oropharynx, hypopharynx, and larynx), with different etiology (tobacco and/or HPV infection), clinical behavior, and response to treatment [2]. In addition, despite the latest developments in diagnosis and treatment, the prognosis has only modestly improved over recent decades.

Immune evasion is an important hallmark of cancer [3]. Several mechanisms have been identified, with multiple immune checkpoints exploited by tumor cells to induce the exhaustion of effector T cells in the tumor microenvironment (TME) [4]. These immune checkpoints become key targets for anti-cancer immunotherapy to redirect host immune responses and match tumor adaptability [4]. The best-studied mechanism of immune evasion is the expression by tumor cells of programmed cell death ligand 1 (PD-L1), which interacts with programmed cell death protein (PD-1) on cytotoxic T lymphocytes, resulting in a reduced T-cell activation and proliferation [4]. A PD-1 receptor is a negative regulator of T-cells that binds to PD-L1, which is a surface glycoprotein primarily expressed by myeloid dendritic cells, activated T cells, and some nonhematopoietic tissues and is a major mediator of immunosuppression. Blockade of PD-1/PD-L1 pathway members has demonstrated efficacy in the treatment of several cancers [4]. Two anti-PD-1 monoclonal antibodies (nivolumab and pembrolizumab) have been approved by the US Food and Drug Administration (FDA) for the treatment of patients with recurrent/metastatic (R/M) HNSCC [5]. PD-L1 expression has been proposed as a predictive biomarker for response to immunotherapy [5]. In addition, tumors could be categorized by gene expression to have T-cell-inflamed or non-inflamed TME, and this phenotype has been correlated to the response to immune checkpoint blockade [6].

β-catenin is a multi-functional protein and central component of the canonical WNT signaling pathway. In addition, β-catenin forms a complex with E-cadherin at the epithelial adherens junction that is critical for cell–cell contacts and tissue remodeling [7]. β-catenin could play a dual role in tumorigenesis, depending on its subcellular localization: anti-oncogenic, as a component of the cadherin-catenin complexes (at the cell membrane), and pro-oncogenic, as a signaling factor (in the nucleus). WNT signaling inhibits β-catenin proteasomal degradation, leading to its cytoplasmic accumulation [7]. Subsequent nuclear translocation of β-catenin allows its binding to the family of T-cell factor/lymphoid enhancer factor (TCF) transcription factors, thereby inducing the expression of target genes, such as cyclin D1, AXIN2, and many others depending on the cellular/tissue context [7,8]. Activation of the WNT signaling has been related to malignant transformation, and β-catenin has been involved as an oncogene in the pathogenesis of several malignant tumors [9]. Moreover, WNT/β-catenin signaling has been implicated in tumor immune evasion [10,11]. WNT/β-catenin signaling in tumor cells was the first somatic alteration associated with the non-T-cell-inflamed TME in a metastatic human cutaneous melanoma model [12]. Furthermore, according to a recent TCGA analysis, tumor-cell intrinsic activation of WNT/β-catenin signaling could contribute to a non-T-cell-inflamed TME in multiple tumor types (including HNSCC) [13]. Therefore, WNT/β-catenin signaling emerges as a mechanism of immune exclusion by tumor cells that has been increasingly and widely recognized in multiple cancers [14].

On this basis, the aim of this study was to investigate more deeply the relationship between tumor-intrinsic WNT/β-catenin signaling and a non-T-cell-inflamed TME and the clinical and prognostic implications in HNSCC by analyzing a large homogeneous cohort of 372 surgically treated HPV-negative HNSCC patients.

## 2. Results

The characteristics of the patients selected for study are summarized in Supplementary Table S1. Only fourteen patients were women, and the mean age was 58.6 years. All the patients had a single primary tumor and did not receive any treatment prior to surgery. Sixty-two percent (232) of the 372 patients received adjuvant radiotherapy.

### 2.1. Evaluation of PD-L1 Expression and CD8+ T-Cell Infiltration in HNSCC Patient Samples

**Figure 1:**
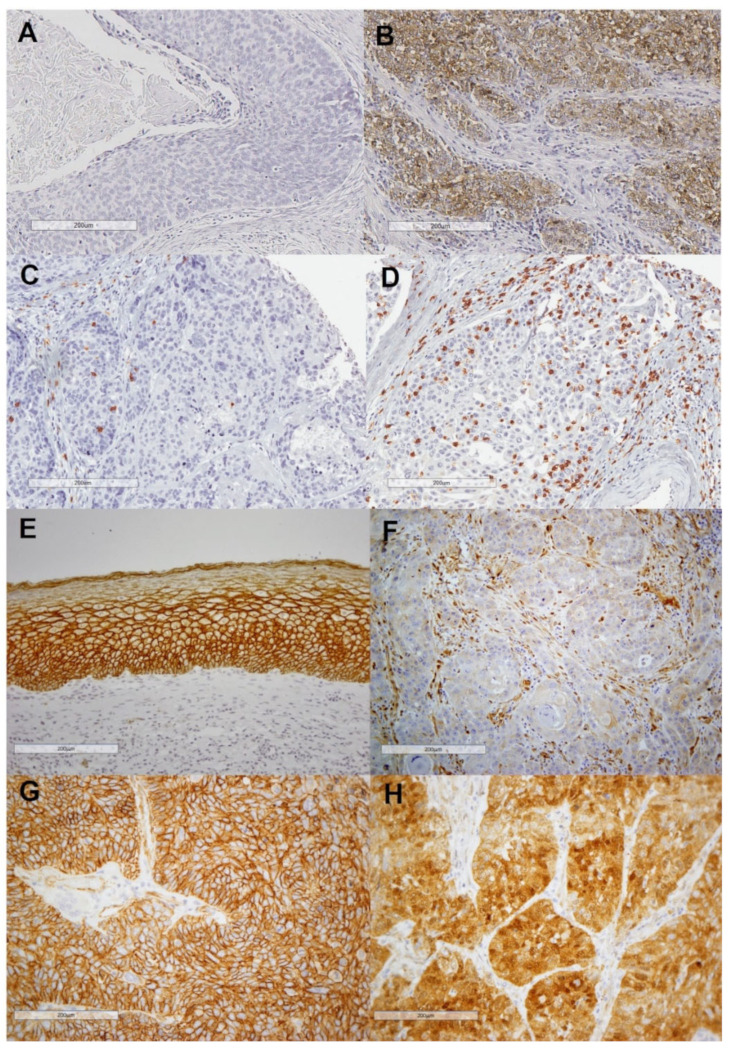
Representative examples of negative (**A**) and positive (**B**) PD-L1 expression; low (**C**) and high (**D**) CD8+ T-cells infiltration; expression of β-catenin in normal epithelium (**E**) and absence of expression of β-catenin (**F**), membrane expression of β-catenin (**G**), and nuclear expression of β-catenin (**H**) in carcinomas. Original magnification ×200.

PD-L1 immunostaining could be assessed in 349 out of 372 patients; 103 (29.5%) of 349 cases showed positive PD-L1 staining in tumor cells (Figure 1A,B). A total of 92 cases (26%) exhibited positive PD-L1 staining in stromal immune cells, and 37 of them were with negative PD-L1 expression in the tumor. There was a positive significant correlation between tumoral and stromal PD-L1 expression (Spearman’s Rho coefficient = 0.373, *p* < 0.001). Then, the PD-L1 tumor proportion score was positive (TPS ≥ 1%) in 103 cases (29.5%), and the PD-L1 combined proportion score was positive (CPS ≥ 1%) in 140 cases (40%). In addition, CD8+ TIL immunostaining could be analyzed in 337 HNSCC samples; the mean number of CD8+ TILs per mm^2^ was 275 (median = 182, range 4–2670). Representative examples of tumors with high and low densities of CD8+ TILs are shown in Figure 1C,D.

### 2.2. Immunohistochemical Analysis of β-Catenin Expression in HNSCC Patient Samples

Strong membrane β-catenin staining was observed in the normal epithelium used as a positive control (IRS = 6; Figure 1E). β-catenin immunostaining was evaluable in 349 HNSCC samples. Membrane-β-catenin expression was detected in 333 tumor cases, showing weaker staining than the normal squamous epithelium, with a mean IRS score of 3.9 ± 1.9 (median 4). Nuclear β-catenin was observed in 50 cases (14.3%). It is noteworthy that these cases generally showed negative or very weak membrane expression. Thus, the mean membrane IRS score for the cases with positive nuclear β-catenin expression was 2.1 compared to a mean IRS of 4.3 for the cases with negative nuclear β-catenin expression (ANOVA *p* < 0.001). Representative examples of negative and positive membrane and nuclear β-catenin staining are shown in Figure 1F–H.

### 2.3. Correlations between β-Catenin Expression in Tumors, PD-L1 Expression, and TIL Infiltration

We found that low levels (IRS < 3) of membrane β-catenin were significantly associated with a lower CD8+ mean density (ANOVA *p* = 0.008; Table 1). Moreover, there was a positive correlation between the IRS scores for membrane β-catenin expression and CD8+ TILs density (Spearman r = 0.179, *p* = 0.001). In addition, low CD8+ TIL density was observed in tumors harboring positive nuclear β-catenin expression (ANOVA *p* = 0.01; Table 1).

There was a positive correlation between PD-L1 CPS positivity and membrane β-catenin expression, and a negative correlation with nuclear β-catenin; however, differences did not reach statistical significance (Spearman Rho = 0.074, *p* = 0.17, and Spearman Rho = −0.076, *p* = 0.16, respectively).

We next assessed the associations of β-catenin expression with the type of immune tumor microenvironment according to the classification described by Teng et al. [15]. Tumors harboring low membrane β-catenin or positive nuclear expression of β-catenin were associated with a type II immune microenvironment (i.e., immunological ignorance), although differences were only statistically significant in the case of membrane β-catenin (Table 2).

### 2.4. Associations with Clinicopathological Characteristics

Positive PD-L1 CPS was significantly associated with a lower pT classification (*p* = 0.046) and an earlier disease stage (*p* = 0.014). Associations between PD-L1 CPS and tumor localization, and pN classification and degree of differentiation were not found (Supplementary Table S2). CD8+ TIL density was significantly lower in stage IV disease (*p* = 0.01); no other associations between CD8+ TIL density and clinicopathological parameters were found (Supplementary Table S2). Positive nuclear β-catenin showed significant associations with tumor site and disease stage, being less frequent in laryngeal tumors (*p* = 0.01) and stage I-III tumors (*p* = 0.03) (Supplementary Table S2). The type of immune TME was significantly correlated with pT classification (*p* = 0.04) and disease stage (*p* = 0.01). Thus, higher pT classification (T3–T4) and advanced stage IV tumors were related to a type II (i.e., immune ignorance) (Supplementary Table S3).

### 2.5. Associations with Patient Survival

**Figure 2:**
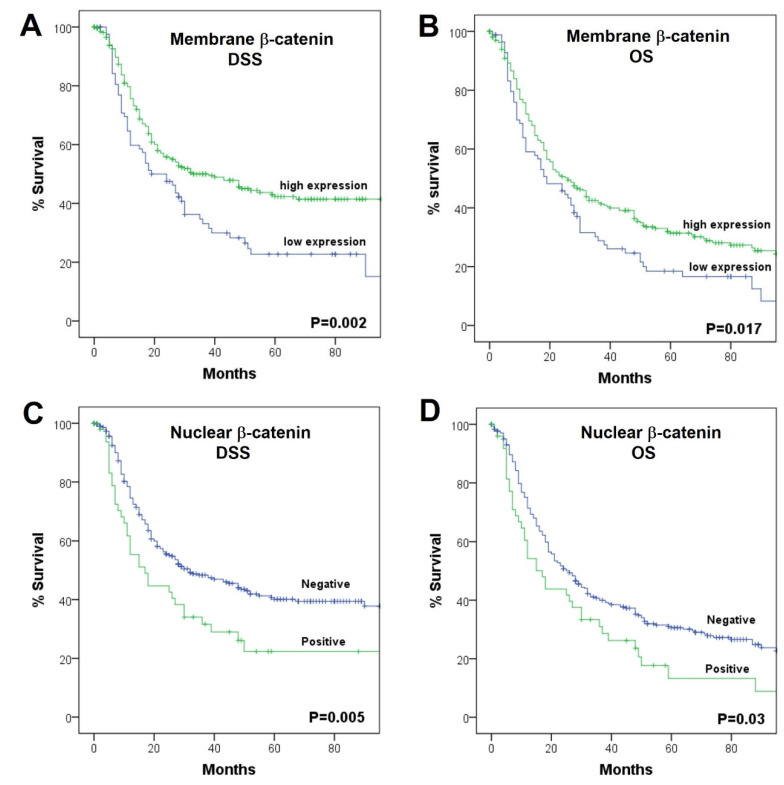
Kaplan–Meier-disease-specific (left) and overall survival (right) curves according to the expression of membrane β-catenin (**A**,**B**) and nuclear β-catenin (**C**,**D**). *p*-values were estimated using the Log-rank test.

**Figure 3:**
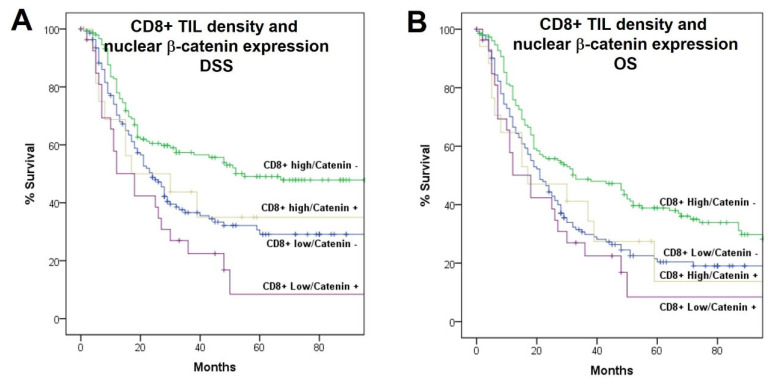
Kaplan–Meier-disease-specific (**A**) and overall survival (**B**) curves according to the combined expression of nuclear β-catenin and CD8+ TIL density.

We previously reported that both PD-L1-CPS-negative expression and a low CD8+ TIL infiltration were significantly associated with a poorer disease-specific survival (DSS) and overall survival (OS) in this HNSCC patient cohort (Supplementary Figure S1), but on multivariate analysis, only CD8+ TIL infiltration was independently associated with survival [12]. In relation to β-catenin expression, patients with low levels (IRS < 3) of membrane β-catenin expression showed a significantly poorer DSS and OS compared to those with tumors harboring high membrane β-catenin (Log-rank, *p* = 0.002 and *p* = 0.017, respectively; Figure 2A,B). Accordingly, positive nuclear β-catenin was also associated with a significantly poorer DSS and OS (Log-rank, *p* = 0.005 and *p* = 0.03, respectively; Figure 2C,D). Combining the infiltration by CD8+ TIL with the nuclear expression of β-catenin, the cases with high CD8+ TIL density and negative expression of nuclear β-catenin had the highest DSS and OS and the cases with low CD8+ TIL density and positive nuclear β-catenin expression had the lowest (*p* < 0.001 and *p* = 0.006, respectively), with the other combinations showing intermediate survival (Figure 3).

Multivariate Cox analysis, including T classification (T1–T2 vs. T3–T4), N classification (N0 vs. N+), degree of differentiation (well-moderately vs. poorly differentiated), CD8+ TIL density (above vs. below the median), and nuclear β-catenin expression (positive vs. negative) showed that the parameters independently associated with a worse DSS were T3–T4 classification (HR = 1.55, 95% CI = 1.11–2.16, *p* = 0.001), N+ classification (HR = 2.44, 95% CI = 1.65–3.1, *p* < 0.001), poor histologic differentiation (HR = 1.29, 95% CI = 1.1–1.52, *p* = 0.003), low CD8+ TIL density (HR = 1.6, 95% CI = 1.19–2.14, *p* = 0.002), and positive nuclear β-catenin expression (HR = 1.47, 95% CI = 1.01–2.16, *p* = 0.04). Similarly, the only parameters independently associated with a worse OS were: T3–T4 classification (HR = 1.48, 95% CI = 1.11–1.98, *p* = 0.007), N+ classification (HR = 2.01, 95% CI = 1.47–2.7, *p* < 0.001), poor histologic differentiation (HR = 1.21, 95% CI = 1.04–1.41, *p* = 0.01), and low CD8+ TIL density (HR = 1.45, 95% CI = 1.12–1.87, *p* = 0.005).

## 3. Discussion

In this work, we show that WNT/β-catenin pathway activation, reflected by positive expression of nuclear β-catenin, is associated with a lower infiltration by TILs and a lower expression of PD-L1, resulting in an immunosuppressive tumor microenvironment. These results are in agreement with recent reports revealing that most tumor types (28/31, 90%) within the TCGA (including HNSCC) showed WNT/β-catenin signaling enrichment in non-T-cell inflamed tumors [13]. Tumor-intrinsic WNT/β-catenin signaling was the first somatic alteration associated with a noninflamed tumor microenvironment in melanoma [12]. Later, other studies exploring the influence of β-catenin activation in the T-cell infiltration in specific tumor types such as colorectal carcinoma [16], non-small lung cancer [17], glioblastoma [18], and adrenocortical carcinoma [19] also showed similar results. However, to the best of our understanding, this is the first study specifically analyzing the relationship between the WNT/β-catenin pathway and the immune tumor microenvironment in HNSCC. Notably, the mean density of CD8+ TILs in HNSCC with increased β-catenin activity was 168 cells/mm^2^ compared to a mean density of 293 cells/mm^2^ in nuclear β-catenin-negative cases (*p* = 0.01), and the cases with nuclear β-catenin expression had a higher proportion of type II tumor microenvironment (defined as immune ignorance) according to the classification reported by Teng et al. [15].

The mechanism of Immune exclusion associated with β-catenin signaling has been described in melanoma [12]. Activated transcription factor 3 (ATF3) expression is induced by β-catenin in melanoma cells, which suppresses C-C motif chemokine ligand 4 (CCL4) gene transcription. The chemokine CCL4 is responsible for the recruitment of CD103+ dendritic cells, which, in turn, activate CD8+ T cells. Therefore, β-catenin signaling results in immune escape due to an insufficient recruitment of dendritic cells [12]. Normal production of CCL4 is restored in the absence of active β-catenin, which results in the activation of CD103+ dendritic cells and the infiltration and priming of CD8+ T cells [10]. Even though cytotoxic T lymphocytes are involved in antigen presentation, a second round of T cell-dendritic cell crosstalk is necessary for T cells to attack tumor cells [20]. Hence, β-catenin activation and subsequent suppression of dendritic cell infiltration might block this crosstalk. Then, tumoral β-catenin signaling may impair antigen presentation and crosstalk, thereby creating a state of low antigenicity that could explain the lower PD-L1 CPS expression in those tumors with positive nuclear β-catenin, as observed in our study and also in other cancers [17]. Consequently, activation of tumor-intrinsic β-catenin might play a role as a resistance mechanism of immune checkpoint blockers [10]. By contrast, it was demonstrated that β-catenin activation was able to induce β-catenin/TCF/LEF complex binding to the CD274 gene promoter and subsequently increase PD-L1 expression in glioblastoma [18]. In addition, the WNT/β-catenin pathway showed no effect on CCL4 gene expression in glioblastoma cells, suggesting that CCL4 is likely not implicated in β-catenin-mediated TIL exclusion in these tumors. Therefore, it seems that WNT/β-catenin signaling could induce immune T cell exclusion from the TME by several mechanisms depending on the tumor type.

We think that the most important finding in our study is the negative association between WNT/β-catenin signaling and CD8+TIL counts. In association with the lower PD-L1 expression, this possibly contributes to the absence of an immune response that confers a poorer prognosis and a potential resistance to immunotherapy for this subset of HNSCC patients. In fact, we have previously shown in this series of patients that a lower CD8+ TIL density [21] was independently associated with a poorer DSS and OS. As a further extension, here, we show that nuclear β-catenin expression (indicative of WNT signaling activation) was significantly associated with a poorer DSS and OS, confirming a more aggressive behavior of the tumors with activation of the WNT/β-catenin pathway. Furthermore, nuclear β-catenin was found to be a significant independent predictor of poor HNSCC prognosis. Additionally, we found that a low expression of membrane β-catenin was associated with a poorer prognosis, which could reflect a more aggressive behavior of tumors with loss of cellular adhesion. 

Activation of WNT/β-catenin signaling seems to constitute an intrinsic mechanism of primary and adaptive resistance of tumor cells to immunotherapy through T cell exclusion. Hence, it emerges as a valuable molecular target to develop novel therapeutic strategies aimed to restore T cell infiltration, which could potentially increase the efficacy of immunotherapy, turning a ‘cold’ tumor into a ‘hot’ tumor (by increasing TIL density) [10]. However, β-catenin signaling is widely utilized by many cell types, and thus, on-target, off-tumor effects may plausibly limit the potential of therapeutically targeting this protein [7].

## 4. Materials and Methods

### 4.1. Patients and Tissue Specimens

Tumor samples from 382 patients with HNSCC who were surgically treated at the Hospital Universitario Central de Asturias (HUCA), Oviedo, Spain, between the years of 1990 and 2009 were retrospectively collected. All experimental procedures were performed in accordance with the Declaration of Helsinki. Formalin-fixed paraffin-embedded (FFPE) tissue samples and data from donors were provided by the Principado de Asturias BioBank (PT17/0015/0023), Oviedo, Spain, integrated in the Spanish National Biobanks Network, and histological diagnosis was confirmed by an experienced pathologist. Protocols were approved by the HUCA Ethics Committee and the Regional CEIm from Principado de Asturias. Informed consent was obtained from all patients. All patients had a single primary tumor and did not receive any treatment before surgery. None of the patients showed distant metastasis at the time of diagnosis. Clinical, demographic, and follow-up data were collected from the medical records.

The HPV status was available for all the patients, which was determined as previously reported [22,23]. To avoid bias, this study only included 372 patients with HPV-negative tumors. The tumors were classified according to the 7th edition of the TNM system of the International Union Against Cancer, Geneve, Switzerland. The mean follow-up for the whole series was 34.6 months (median 21.5 months) and the minimum follow-up for the patients alive at last follow-up was 36 months. Recurrence was defined as tumor relapse in the five first years after treatment at any site: either local or regional recurrence, and/or distant metastasis. 

### 4.2. Tissue Microarray (TMA) Construction

Three 1 mm cylinders selected from representative areas of each individual tumor block were selected to construct TMA blocks, as described previously [22], containing a total of 382 HNSCCs comprising 141 tonsillar, 108 base of tongue, 65 hypopharyngeal, and 68 laryngeal carcinomas. Each TMA also included three cores of normal squamous epithelium from the same anatomical location (i.e., larynx and oro/hypopharynx), as an internal control. Normal tissue samples were obtained from adult male patients (nonsmokers and nondrinkers) who underwent nononcologic surgery (e.g., tonsillectomy and benign vocal cord lesions).

### 4.3. Immunohistochemical Study

The TMA blocks were cut into 3 μm sections and dried on Flex IHC microscope slides (Dako, Santa Clara, CA, USA). After deparaffinization with standard xylene and hydration through graded alcohols into water, antigen retrieval was performed using EnVision Flex Target Retrieval solution, high pH (Dako). Staining was performed using an automatic staining workstation (Dako Autostainer, Dako Cytomation) with the Envision system and the primary antibodies: anti-PD-L1 (clone E1L3N; Cell Signaling Technology, Danvers, MA, USA) at 1:200 dilution, anti-CD8 (clone SP16, Neomarkers, Whaltam, MA, USA) at 1:400 dilution, and anti-β-catenin (BD Biosciences, Franklin Lakes, NJ, USA) at a dilution of 1:200. Counterstaining with hematoxylin was the final step. 

As positive staining controls, we used placenta for PD-L1, and normal tonsil for CD8 and β-catenin expression. Stainings were reviewed and scored blinded to clinical data by two expert pathologists.

For the evaluation of PD-L1 expression in tumor cells, only membrane staining was considered and scored as: (a) negative PD-L1 expression (<1% stained cells), (b) low PD-L1 expression (≥1–<10%), (c) intermediate PD-L1 expression (≥10–<50%), or (d) high PD-L1 expression (≥50%). Tumor proportion score (TPS) was defined as the percentage of PD-L1-positive tumor cells in relation to the total tumor cells, and PD-L1 CPS ≥ 1% was considered positive. Combined proportion score (CPS), which represents the number of all positive PD-L1 cells (tumor cells, lymphocytes, and macrophages) in relation to total tumor cells, was also considered positive if ≥1%. TILs were defined as lymphocytes within the tumor nests, excluding the peritumoral area. Quantification of CD8+ TIL staining (as a marker of cytotoxic T lymphocytes) was performed automatically using ImageJ software; CD8+ T cells were counted in the tumor areas of each core within the TMAs (i.e., three cores per tumor case). The average total number of positive cells in the three tumor cores was expressed in density (number of positive cells per mm^2^). The median value was used as a cut-off point to stratify high versus low density of CD8+ TILs. 

Membrane β-catenin expression was quantified combining the percentage of stained cells (scored from 0 to 3 when positive cells were 0%, 1–10%, 11–50%, and 51–100%, respectively) and staining intensity (0 = negative, 1 = weak/moderate, and 2 = strong). An immunoreactive score (IRS) was calculated by multiplying the quantity and staining intensity scores. IRS scores ≥ 3 were considered as high-membrane-β-catenin expression and IRS scores from 0 to 2 as low membrane expression. Nuclear β-catenin expression was considered positive when ≥10% of tumor cells showed nuclear β-catenin staining.

### 4.4. Statistical Analysis

Comparison between categorical variables was performed using chi-square and Fisher’s exact tests. Kaplan–Meier curves were used for time-to-event analysis. Cox proportional hazards models were used for univariate and multivariate analyses, reporting the hazard ratios (HRs) with a 95% confidence interval (CI), and *p* values. All tests were two-sided. *p*-values of ≤0.05 were considered statistically significant.

## 5. Conclusions

We demonstrate that specifically tumor-intrinsic β-catenin activation in HNSCC is associated with a lower PD-L1 expression and low infiltration of CD8+ T cells, and hence a noninflamed TME and an immune ignorance phenotype. Altogether, these findings suggest that the interference of WNT/β-catenin signaling activity in combination with immune checkpoint blockade might represent a novel strategy to improve the efficacy of immunotherapeutic agents for some subsets of HNSCC patients.

## Figures and Tables

**Table 1:** Correlations between CD8+ TIL density and β-catenin expression in HNSCC patients.

Variable	No. Cases	Mean CD8+ TIL Density (Cells/mm^2^)	*p*
**Membrane β-catenin** - Low (IRS < 3) - High (IRS ≥ 3)	82 260	196 301	0.008
**Nuclear β-catenin** - Negative - Positive	296 48	293 168	0.01

**Table 2:** Correlations between the type of immune tumor microenvironment (TME) and β-catenin expression.

Type of Immune TME	Low Membrane β-Catenin (%)	High Membrane β-Catenin (%)	*p*	Positive Nuclear β-Catenin (%)	Negative Nuclear β-Catenin (%)	*p*
- **Type I** **(PD-L1+/CD8+ high)**	20 (25%)	78 (30%)	0.001	10 (21%)	88 (30%)	0.19
- **Type II** **(PD-L1−/CD8+ low)**	46 (57%)	86 (33.5%)		25 (53%)	107 (37%)	
- **Type III** **(PD-L1+/CD8+ low)**	8 (10%)	32 (12.5%)		5 (11%)	36 (12%)	
- **Type IV** **(PD-L1−/CD8+ high)**	7 (8%)	61 (24%)		7 (15%)	61 (21%)	

## Data Availability

The datasets generated during and/or analyzed during the current study are available from the corresponding author on reasonable request.

## Supplementary Materials

The following supporting information can be downloaded at: https://www.mdpi.com/article/10.3390/ijms231911559/s1. Supplementary Table S1: Clinicopathological characteristics of the 372 HNSCC patients selected for study. Supplementary Table S2: Associations of PD-L1 combined proportion score (CPS), nuclear β-catenin expression, and CD8+ TIL infiltration with the clinicopathological parameters in the studied cohort of 372 HNSCC patients. Supplementary Table S3: Associations between the type of immune tumor microenvironment (TME) and the clinicopathological parameters. Figure S1: Kaplan–Meier disease-specific (left) and overall survival (right) curves according to the combined proportion score (CPS) of PD-L1 (A,B) and CD8+ TIL density (C,D). *p*-values were estimated using the Log-rank test.
