# Supplementary material for: Tumor-Intrinsic Nuclear β-Catenin Associates with an Immune Ignorance Phenotype and a Poorer Prognosis in Head and Neck Squamous Cell Carcinomas
Source: Int J Mol Sci. 2022 Sep 30;23(19):11559. doi: 10.3390/ijms231911559 (PMC9569731; doi:10.3390/ijms231911559)

SUPPLEMENTARY MATERIALS

Tumor-intrinsic nuclear β-catenin associates with an immune ignorance phenotype and a poorer prognosis in head and neck squamous cell carcinomas

Mario Sánchez-Canteli [1,2,*], Luis Juesas [1], Irati Garmendia [2,3], María Otero-Rosales [1], Alfonso Calvo [2,3], Mónica Álvarez-Fernández [1], Aurora Astudillo [4], Luis M Montuenga [2,3], Juana M. García-Pedrero [1,2] and Juan P. Rodrigo [1,2,*]

[1] Department of Otolaryngology, Hospital Universitario Central de Asturias and Instituto de Investigación Sanitaria del Principado de Asturias (ISPA), Instituto Universitario de Oncología del Principado de Asturias, University of Oviedo, 33011, Oviedo, Spain.

[2] CIBERONC, 28029, Madrid, Spain.

[3] Program in Solid Tumors, Center for Applied Medical Research (CIMA), Department of Pathology, Anatomy and Physiology, University of Navarra and Navarra's Health Research Institute (IDISNA), 31008, Pamplona, Spain.

[4] Tumor Biobank. Instituto de Investigación Sanitaria del Principado de Asturias (ISPA), Instituto Universitario de Oncología del Principado de Asturias, University of Oviedo, 33011, Oviedo, Spain.

***** Correspondence: jprodrigo@uniovi.es (JPR); mariosanchezcanteli@gmail.com (MSC)

Supplementary Table S1. Clinicopathological characteristics of the 372 HNSCC patients selected for study.

Characteristic	No. Patients (%)
**Age, mean (range)**	58.6 (30–86 years)
**Tobacco**	
Non-smokers	12 (3.5)
Moderate (1-50 packs-year)	207 (55.5)
Heavy (>50 packs-year)	153 (41)
**Alcohol**	
Non-drinkers	37 (10)
Drinkers	335 (90)
**Location**	
Oropharynx	241 (65)
Hypopharynx	64 (17)
Larynx	67 (18)
**pT classification**	
T1	38 (10)
T2	77 (21)
T3	125 (34)
T4	132 (35)
**pN classification**	
N0	103 (28)
N1	46 (12)
N2	183 (49)
N3	40 (11)
**Stage**	
I	20 (5)
II	24 (6)
III	64 (17)
IV	264 (71)
**Degree of differentiation**	
Well-differentiated	147 (39)
Moderately differentiated	148 (40)
Poorly differentiated	77 (21)
**Total**	372

Supplementary Table S2. Associations of PD-L1 combined proportion score (CPS), nuclear β-catenin expression and CD8+ TIL infiltration with the clinicopathological parameters in the studied cohort of 372 HNSCC patients.

**Parameter**	**Negative PD-L1 CPS (%)**	**Positive PD-L1 CPS (%)**	***P***	**Negative nuclear β-catenin (%)**	**Positive nuclear β-catenin (%)**	***P***	**Low CD8+ TIL (%)**	**High CD8+ TIL (%)**	***P***
Localization			0.74			*0.01*			0.8
- Oropharynx	143 (60%)	96 (40%)		200 (83%)	41 (17%)		119 (51%)	115 (49%)	
- Hypopharynx	31 (54%)	26 (46%)		49 (87%)	7 (13%)		28 (48%)	30 (52%)	
- Larynx	35 (60%)	23 (40%)		57 (95%)	3 (5%)		31 (53%)	27 (47%)	
pT classification			*0.04*			0.6			0.4
- T1-T2	54 (51%)	52 (49%)		91 (84%)	17 (16%)		49 (47%)	55 (53%)	
- T3-T4	155 (62%)	93 (38%)		215 (86%)	34 (14%)		129 (52%)	117 (48%)	
pN classification			0.32			0.35			0.27
- N0	50 (54%)	42 (46%)		85 (88%)	11 (12%)		43 (46%)	51 (54%)	
- N1-3	159 (61%)	103 (39%)		221 (85%)	40 (15%)		135 (53%)	121 (47%)	
Disease stage			*0.01*			*0.03*			*0.01*
- I-II	19 (50%)	19 (50%)		35 (90%)	4 (10%)		15 (39%)	23 (61%)	
- III	28 (45%)	34 (55%)		58 (95%)	3 (5%)		23 (38%)	38 (62%)	
- IV	162 (64%)	92 (36%)		213 (83%)	44 (17%)		140 (56%)	111 (44%)	
Degree of differentiation			0.57			0.06			0.18
- G1	84 (60%)	57 (40%)		121 (86%)	20 (14%)		70 (50%)	69 (50%)	
- G2	84 (61%)	53 (39%)		124 (90%)	14 (10%)		76 (56%)	60 (44%)	
- G3	41 (54%)	35 (46%)		60 (78%)	17 (22%)		32 (43%)	43 (57%)	

Supplementary Table S3. Associations between the type of immune tumor microenvironment (TME) and the clinicopathological parameters.

**Parameter**	**Type I TME (%)**	**Type II TME (%)**	**Type III TME (%)**	**Type IV TME (%)**	***P***
Localization					0.75
- Oropharynx	63 (27%)	88 (38%)	30 (13%)	52 (22%)	
- Hypopharynx	18 (33%)	19 (34%)	7 (13%)	11 (20%)	
- Larynx	17 (31%)	25 (45%)	5 (9%)	8 (15%)	
pT classification					
- T1-T2	33 (33%)	29 (29%)	18 (18%)	20 (20%)	*0.04*
- T3-T4	65 (27%)	103 (42%)	24 (10%)	51 (21%)	
pN classification					0.42
- N0	29 (33%)	28 (31%)	13 (15%)	19 (21%)	
- N1-3	69 (27%)	104 (41%)	29 (11%)	52 (20%)	
Disease stage					
- I-II	12 (34%)	7 (20%)	7 (20%)	9 (26%)	*0.01*
- III	24 (40%)	14 (23%)	9 (15%)	13 (22%)	
- IV	62 (25%)	111 (45%)	26 (10%)	49 (20%)	
Degree of differentiation					0.12
- G1	38 (28%)	52 (38%)	18 (13%)	29 (21%)	
- G2	30 (23%)	54 (41%)	20 (15%)	29 (22%)	
- G3	30 (41%)	26 (36%)	4 (5%)	13 (18%)	

Supplementary Figure S1. Kaplan-Meier disease specific (*left*) and overall survival (*right*) curves according to the combined proportion score (CPS) of PD-L1 (A, B) and CD8+ TIL density (C, D). *P* values were estimated using the Log-rank test

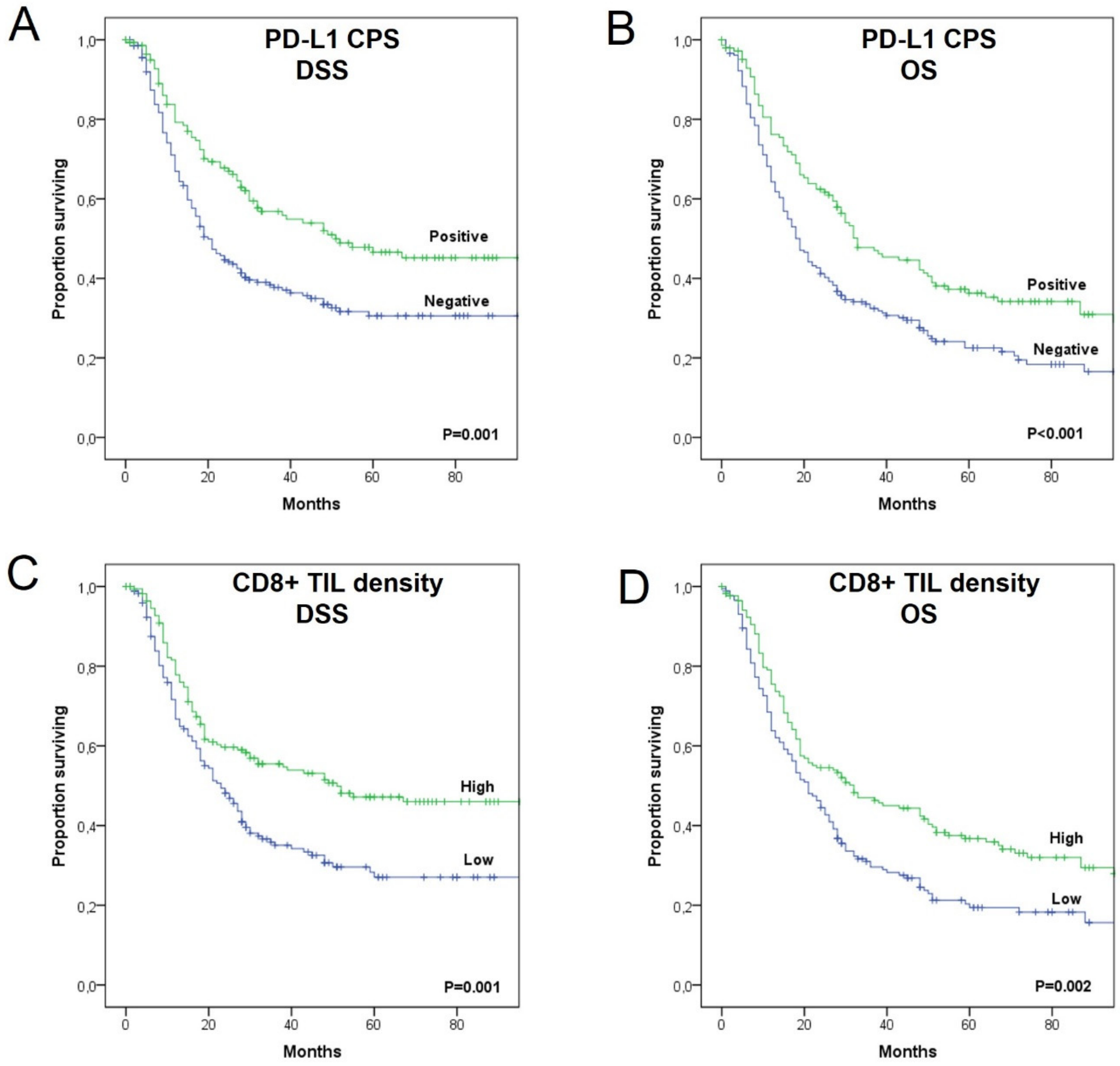

Supplement: Supplementary file 1 [file ijms-23-11559-s001.zip › ijms-1914848-supplementary.pdf]
